# Eosinophil Inversely Associates with Type 2 Diabetes and Insulin Resistance in Chinese Adults

**DOI:** 10.1371/journal.pone.0067613

**Published:** 2013-07-22

**Authors:** Liying Zhu, Tingwei Su, Min Xu, Yu Xu, Mian Li, Tiange Wang, Jichao Sun, Jie Zhang, Baihui Xu, Jieli Lu, Yufang Bi, Weiqing Wang, Yiping Xu

**Affiliations:** 1 Key Laboratory for Endocrine and Metabolic Diseases of Ministry of Health, Rui-Jin Hospital, Shanghai Jiao-Tong University School of Medicine, E-Institute of Shanghai Universities, Shanghai, China; 2 Shanghai Clinical Center for Endocrine and Metabolic Diseases, Shanghai Institute of Endocrine and Metabolic Diseases, Department of Endocrinology and Metabolism, Rui-Jin Hospital, Shanghai Jiao-Tong University School of Medicine, Shanghai, China; 3 Department of Research and Development, Rui-Jin Hospital, Shanghai Jiao-Tong University School of Medicine, Shanghai, China; 4 Institute of Health Sciences, Shanghai Institutes for Biological Sciences, Chinese Academy of Sciences and Shanghai Jiao-Tong University School of Medicine, Shanghai, China; Rui-Jin Hospital, China

## Abstract

**Context:**

Limited population-based study focused on relationship between eosinophil and type 2 diabetes (T2D).

**Objectives:**

We aimed to evaluate the relationship between peripheral eosinophil percentage and glucose metabolism and insulin resistance in a large sample size of Chinese population aged 40 and older.

**Design and Methods:**

A cross-sectional study was performed among 9,111 Chinese adults including 3,561 men and 5,550 women. The glucose metabolism status was confirmed by 75-g oral glucose tolerance test. Homeostasis model assessment of insulin resistance index and serum insulin levels were used to evaluate insulin resistance. Homeostasis model assessment-B was used to evaluate β cell function.

**Results:**

The average age of participants was 58.5 years. The prevalence of T2D decreased across the tertiles of eosinophil percentage (21.3%, 18.2% and 16.9%, P<0.0001). Each one tertile increase of eosinophil percentage inversely associated with risk of T2D when referred not only to normal glucose tolerance (NGT) (odds ratio (OR) 0.81, 95% CI 0.76–0.87, P< 0.0001), but also to impaired glucose regulation (OR 0.89, 95% CI 0.83–0.97, P = 0.006), respectively, after adjustment for the confounding factors. Compared with the first tertile, the third tertile of eosinophil percentage associated with a 23% decrease of insulin resistance in NGT participants after full adjustments (P = 0.005). Each 1-standard deviation of increment of eosinophil percentage associated with a 37% decrease of insulin resistance (P = 0.005).

**Conclusions:**

Higher peripheral eosinophil percentage was associated with decreased risk of T2D. The inverse relation to insulin resistance was detected in NGT participants.

## Introduction

Immune response and metabolic regulation are highly integrated and interacted with each other in keeping the proper physical function. This interface can be viewed as a central homeostatic mechanism, dysfunction of which can lead to a cluster of chronic metabolic disorders, particularly obesity, type 2 diabetes (T2D) and cardiovascular diseases [1]. A chronic low-grade activation of the immune system, which can be detected by an increase in number of markers including white blood cell (WBC) count and cytokines etc [2]–[5], may play a role in the pathogenesis of T2D [6]. Eosinophil, one type of WBC, becomes active when people have certain allergic diseases and infections. One previous study reported that patients with asthma rarely developed diabetes mellitus [7]. Recently, a *Science* paper has reported in an animal study that helminth-induced adipose tissue eosinophilia enhanced glucose tolerance. It was hypothesized that eosinophil might take an active part in the pathogenesis of T2D [8]. In the present study, we investigated the association of peripheral eosinophil percentage with T2D, impaired glucose regulation and insulin resistance in a community-based Chinese population.

## Research Design and Methods

### Study population

This cross-sectional investigation was conducted among 10,375 participants aged 40 years or older from a community-based glucose survey in Jiading District, Shanghai, China, from March to August, 2010. Briefly, a total of 10,569 inhabitants aged ≥40 years were invited by telephone or door-to-door visit to participate in this study during the recruiting phase. Then, 10,375 residents agreed to take part in the present study (response rate 98.2%). Participants meeting the following criteria were excluded: 1) subjects with missing values of biochemical measurements (n = 205); 2) subjects who had autoimmune diseases and respiratory diseases or with the treatment of corticoid, insulin and hypoglycemic drugs (n = 87); and 3) subjects having WBC count out of the normal range ((4.0–10.0)×10^9^ cell/L) (n = 1144). At last, 9,111 participants were included in the present study. The Institutional Review Board of Rui-jin Hospital, Shanghai Jiao-Tong University School of Medicine, approved the study protocol, and all the participants provided written informed consents. Completion of the questionnaire and collection of a blood sample were considered to imply informed consent.

### Clinical and biochemical measurements

A standard questionnaire was used to collect the information about health status, medications and lifestyles. The smoking or alcohol consumption habit was defined as never, current (smoking or consuming alcohol regularly in the past 6 months), or former (cessation of smoking or alcohol consumption for more than 6 months).

Body height, body weight, and waist circumferences (WC) were measured by the same trained panel. Weight was measured to the nearest 0.1 kg, and height was recorded to the nearest 0.1 cm while participants were wearing lightweight clothing and no shoes. WC was measured to the nearest 0.1 cm at the umbilical level with the participant in a standing position. Blood pressure was measured at the non-dominant arm in a seated position after a ten-min rest using an automated electronic device (OMRON Model HEM-752 FUZZY, Omron Company, Dalian, China). Three measurements were taken in one minute apart and the average of the three measurements was used in analysis.

Venous blood samples were collected after an overnight fasting. Peripheral leukocyte analyses included total leukocyte count and eosinophil percentage using an automated cell counter (Hematology analyzer 120, ABX, France). Blood glucose, including fasting plasma glucose (FPG) and 2-hour post-loading plasma glucose after 75-g oral glucose tolerance test (2 h OGTT PPG) was measured by using glucose oxidase method on an autoanalyzer (Modular P800; Roche, Basel, Switzerland). Fasting serum insulin, triglycerides (TG), total cholesterol (TC), high-density lipoprotein cholesterol (HDL) and low-density lipoprotein cholesterol (LDL) were measured using chemiluminescence methods on the autoanalyzer (Modular E170; Roche). Glycosylated hemoglobin A1c (HbA1c) level was measured by high performance liquid chromatography (HPLC, BIO-RAD Company, USA).

### Definitions

T2D was diagnosed according to the 1999 World Health Organization (WHO) criteria (FPG≥7.0 mmol/l and/or 2 h OGTT PPG≥11.1 mmol/l, or taking anti-diabetic agents). Impaired glucose regulation (IGR) was defined as impaired fasting glucose (IFG, FPG≥6.1 and <7.0 mmol/l) and/or impaired glucose tolerance (IGT, 2 h OGTT PPG≥7.8 and <11.1 mmol/l). FPG<6.1 mmol/l and 2 h OGTT PPG<11.1 mmol/l and without any anti-diabetic therapy were defined as normal glucose tolerance (NGT). Body mass index (BMI) was calculated as body weight in kilograms divided by height squared in meters (kg/m^2^). The insulin resistance index [homeostasis model assessment of insulin resistance (HOMA-IR)] was calculated as fasting insulin (mU/l)×fasting glucose (mmol/l)/22.5 [9].

### Statistical analysis

SAS version 9.2 (SAS Institute, Cary, NC, USA) was used for database management and statistical analysis. Eosinophil percentage, WBC, FPG, 2 h OGTT PPG, fasting serum insulin, TG and HOMA-IR were normalized by logarithmic transformation before comparisons because of the skewed distributions. Descriptive data were shown as means ± SD (unless indicated otherwise). Comparisons of means and proportions were performed using the analysis of variance (ANOVA) and χ^2^ tests respectively. The subjects were categorized into tertiles of eosinophil percentage (eosinophil percentage ≤1.7%, 1.8–2.7%, >2.7%). Comparisons between groups were performed using ANOVA. Pearson correlation analysis was performed to evaluate the correlation between peripheral eosinophil percentage and clinical factors in different glucose metabolism status. We used the multinomial logistic regression to evaluate the association of eosinophil percentage with risk of present T2D and IGR. The multivariate logistic regression was used to study the association of eosinophil percentage with risk of insulin resistance and elevated fasting serum insulin. In our study, subjects with equal to and/or more than the highest quartile of HOMA-IR, 1.98 in NGT group, 2.80 in IGR and 4.48 in T2D were defined as having insulin resistance. Subjects with equal to and/or more than the highest quartile of fasting serum insulin, 8.90 mIU/l in NGT, 11.50 mIU/l in IGR and 13.50 mIU/l in T2D were defined as having elevated levels of fasting serum insulin. Factorial design was used to evaluate the interaction between WBC count and eosinophil percentage on insulin resistance. All statistical tests were two-tailed and P<0.05 was considered statistically significantly different.

## Results

In this cross-sectional analysis of 9,111 subjects, 60.9% were women. The mean age was 58.5±9.7 years. The distribution of eosinophil percentage was positively skewed with a median (inter-quartile range) of 2.1(1.6–3.1)%. The medians (inter-quartile range) of eosinophil percentage were 2.4(1.7–3.6)% in men and 2.0(1.5–2.8)% in women (P<0.0001).

The clinical characteristics of the participants according to the tertiles of eosinophil percentage are shown in Table 1. FPG, 2 h OGTT PPG and HDL decreased from the first tertile to the third tertile (all P≤0.02). However, there is no statistical difference for fasting serum insulin, HOMA-IR, TG, TC and LDL among the three groups. The prevalence of T2D decreased across the eosinophil percentage tertiles (21.3%, 18.2% and 16.9%, respectively, P for trend<0.0001). The prevalence of IGR showed the same trend (P<0.0001) (Table 1).

**Table 1:** Clinical characteristics of the study population according to tertiles of eosinophil percentage.

	Tertiles of eosinophil percentage			P value
	1^st^tertile	2^nd^tertile	3^rd^tertile	
Range of EOS%	≤1.7	1.8–2.7	>2.7	
*n*	3099	3061	2951	
Age (years)	57.8±9.6	58.6±9.5	59.0±9.9	<0.0001
Sex (%Female)	70.4	61.7	50.2	<0.0001
Current smokers, *n* (%)	450 (14.5)	596 (19.5)	827 (28.0)	<0.0001
Current drinkers, *n* (%)	251 (8.1)	295 (9.6)	374 (12.7)	<0.0001
BMI (kg/m^2^)	25.0±3.2	25.5±3.2	25.3±3.3	<0.0001
SBP (mmHg)	141.6±20.1	142.0±19.9	140.8±19.8	0.004
WC (cm)	82.0±8.8	83.6±8.9	83.8±9.0	<0.0001
Fasting plasma glucose (mmol/l)	5.25 (4.83–5.89)	5.19 (4.79–5.76)	5.15 (4.77–5.67)	<0.0001
PP 2 h plasma glucose (mmol/l)	7.25 (5.88–9.80)	7.03 (5.73–9.22)	6.93 (5.53–8.89)	<0.0001
HbA1c (%)	5.8±1.0	5.8±0.9	5.8±0.9	0.02
Fasting serum insulin (mU/l)	7.18 (4.97–10.50)	7.20 (5.00–10.35)	6.89 (4.64–9.90)	0.31
HOMA-IR	1.69 (1.14–2.64)	1.70 (1.14–2.56)	1.59 (1.05–2.47)	0.28
White blood cell count (×10∧9/l)	5.8 (5.0–6.8)	5.8 (5.0–6.7)	5.7 (4.9–6.6)	<0.0001
Triglycerides (mmol/l)	1.38 (0.98–1.98)	1.42 (1.01–2.00)	1.40 (1.00–2.00)	0.59
Total cholesterol (mmol/l)	5.38±1.02	5.38±1.03	5.32±1.01	0.50
HDL cholesterol (mmol/l)	1.34±0.32	1.31±0.31	1.30±0.31	0.01
LDL cholesterol (mmol/l)	3.21±0.88	3.23±0.85	3.19±0.87	0.22
IGR, *n* (%)	757(24.4)	737(24.1)	661(22.4)	<0.0001
Diabetes, *n* (%)	659(21.3)	558(18.2)	499(16.9)	<0.0001

Data are expressed as mean ± SD or median (interquartile range) or number (proportion).

*P values* were calculated by using ANCOVA with adjustment for age, sex, current smoking and current drinking, or chi-square analysis for category variables.

EOS, eosinophil; WC, waist circumference; SBP, systolic blood pressure; PP, postprandial; HOMA-IR, insulin resistance assessed by homeostasis model assessment; HDL cholesterol, high-density lipoprotein-cholesterol; IGR, impaired glucose regulation.

Simple correlation analysis showed that eosinophil percentage was positively correlated with age, current smoking, current drinking and WC (all P<0.0001), and negatively correlated with FPG, 2 h OGTT PPG, HbA1c, fasting serum insulin, HOMA-IR and HDL in NGT (all P≤0.03). However, eosinophil percentage was negatively correlated with FPG, HbA1c and HDL in IGR (all P≤0.004). In T2D, the correlation between eosinophil percentage and FPG, 2 h OGTT PPG, fasting serum insulin and HOMA-IR disappeared (all P>0.05).

Increase of peripheral eosinophil percentage was significantly associated with decreased risk of T2D when referred not only to NGT (OR 0.81 [95%CI 0.76–0.87]; P<0.0001), but also to IGR (OR 0.89 [95%CI 0.83–0.97]; P = 0.006), after adjustment for age, sex, current smoking, current drinking (Model 2, Table 2). Further adjusted for BMI, WC, SBP, TC, logTG, HDL, LDL, logWBC, logHOMA-IR based on Model 2, the association between eosinophil percentage and T2D did not radically changed. The subjects with a higher peripheral eosinophil percentage associated with a lower risk of IGR (OR 0.91 [95% CI 0.85–0.97]; P = 0.008) after the full adjustment (Table 2).

**Table 2:** The risk of present impaired glucose regulation and type 2 diabetes in relation to each tertile increase of plasma eosinophil percentage by using multinomial logit models.

	NGT(No = 5240)	IGR(N = 2155)		T2D^1^(N = 1716)		T2D^2^(N = 1716)	
		OR (95%CI)	P for trend	OR (95%CI)	P for trend	OR (95%CI)	P for trend
Model 1	1.00	0.91 (0.86–0.97)	0.003	0.84 (0.79–0.90)	<0.0001	0.93 (0.86–1.00)	0.05
Model 2	1.00	0.91 (0.85–0.97)	0.003	0.81 (0.76–0.87)	<0.0001	0.89 (0.83–0.97)	0.006
Model 3	1.00	0.91 (0.85–0.97)	0.008	0.82 (0.76–0.89)	<0.0001	0.91 (0.83–0.98)	0.02

Data are odds ratios (ORs, 95% confidential interval).

Model 1, unadjusted;

Model 2, adjusted for age, sex, current smoking and current drinking;

Model 3, based on model 2 further adjusted for BMI, WC, SBP, TC, logTG, HDL-C, LDL-C, logWBC, logHOMA-IR;

T2D^1^, referenced to normal glucose tolerance (NGT);

T2D^2^, referenced to impaired glucose regulation (IGR).

**Figure 1:**
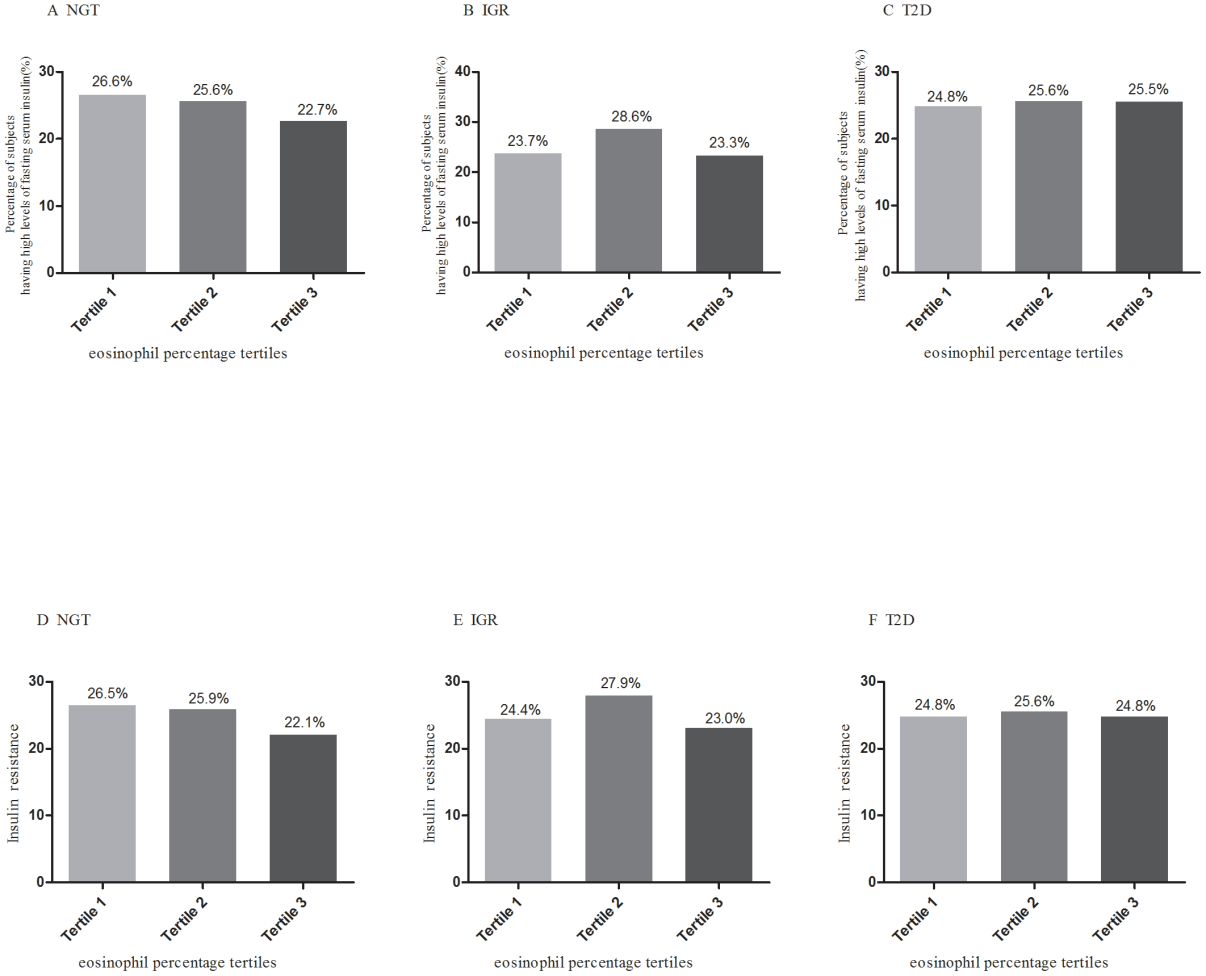
Percentages of elevated fasting serum insulin and insulin resistance across tertiles of eosinophil percentage among NGT, IGR and T2D. (A): Elevated fasting serum insulin in NGT; (B): Elevated fasting serum insulin in IGR; (C): Elevated fasting serum insulin in T2D; (D): Insulin resistance in NGT; (E): Insulin resistance in IGR; (F): Insulin resistance T2D. Number above every bar showed the percentages of the subjects having elevated levels of fasting serum insulin and insulin resistance.

The percentage of elevated fasting serum insulin in the 3^rd^ tertile of eosinophil percentage was bigger than that in the 1^st^ tertile (22.7% vs.26.6%, P = 0.03, Fig. 1, panel A), with the percent of insulin resistance the same (22.1% vs.26.5%, P = 0.01, Fig. 1, panel D). The logistic regression analysis showed that higher eosinophil percentage was associated with decreased risk of elevated fasting serum insulin and insulin resistance (Table 3) with the first tertile of eosinophil percentage as reference after adjustment for the full confounders in NGT. But among participants with IGR and T2D, the similar results cannot be found (Table 3, Fig. 1). Each 1-SD increase of eosinophil percentage was in relation to a 34% decrease of elevated fasting serum insulin (OR 0.66 [95% CI 0.47–0.92]; P = 0.01) and a 37% decrease of insulin resistance (OR 0.63 [95% CI 0.45–0.87]; P = 0.005), respectively in NGT.

**Table 3:** The risk of present insulin resistance in relation to increase tertile of eosinophil percentage among participants with different glucose metabolism status.

	Eosinophil percentage (%)		Elevated fasting serum insulin		Insulin resistance	
			OR (95%CI)	P value	OR (95%CI)	P value
NGT(n = 5240)	Tertiles	T1(≤1.8)	1.00		1.00	
		T2(1.8–2.8)	0.87(0.73–1.03)	0.84	0.90(0.75–1.07)	0.78
		T3(>2.8)	0.78(0.65–0.93)	0.03	0.77(0.64–0.92)	0.01
	1-SD increment		0.66(0.47–0.92)	0.01	0.63(0.45–0.87)	0.005
IGR(n = 2155)	Tertiles	T1(≤1.7)	1.00		1.00	
		T2(1.7–2.6)	1.09(0.83–1.43)	0.26	1.00(0.76–1.31)	0.51
		T3(>2.6)	0.90(0.68–1.20)	0.24	0.85(0.64–1.12)	0.18
	1-SD increment		0.83(0.49–1.41)	0.49	0.72(0.43–1.22)	0.23
Type 2 diabetes(n = 1716)	Tertiles	T1(≤1.6)	1.00		1.00	
		T2(1.6–2.5)	1.00(0.74–1.35)	0.89	0.98(0.73–1.32)	0.66
		T3(>2.5)	0.96(0.71–1.31)	0.78	0.86(0.63–1.16)	0.28
	1-SD increment		1.12(0.63–1.98)	0.70	0.87(0.49–1.53)	0.62

Data are odds ratios (ORs, 95% confidential interval).

Subjects with equal to and/or more than the highest quartile of fasting serum insulin, 8.90 mU/l in NGT group, 11.50 mU/l in IGR and 13.50 mU/l inT2D were defined as having high levels of fasting serum insulin. Subjects with equal to and/or more than the highest quartile of HOMA-IR index, 1.98 in NGT, 2.80 in IGR and 4.48 in T2D were defined as having insulin resistance.

ORs were adjusted for age, sex, current smoking, current drinking, BMI, WC, SBP, logTG, total cholesterol, HDL-C, LDL-C.

Also, we studied the relationship between eosinophil percentage and HOMA-B, but there was no statistical significance. The eosinophil percentage may associate with insulin sensitivity (r = 0.02, P<0.0001) rather than insulin secretion (r = 0.01, P = 0.25).

## Discussion

To the best of our knowledge, this is the first report on the association between peripheral eosinophil percentage and impaired glucose metabolism and insulin resistance in a large population. We found that high eosinophil percentage associated with a decreased risk of T2D and IGR, and lower risk of insulin resistance in NGT participants.

A meta-analysis about WBC count and T2D showed that total WBC count was significantly associated with T2D, even WBC count in the normal range [10]. Vozarova B et al found that a high WBC count was associated with a decline in insulin sensitivity [6]. In the present study, we used the factorial design to do the analysis of variance and the result showed no interaction between WBC and eosinophil percentage on insulin resistance.

Our findings are in line with several previous experimental studies that showed a relationship between diabetes and eosinophil. High risk of diabetes was associated with decreased allergic inflammation which can be reflected by eosinophil [11]–[13]. It has been found that antigen-evoked eosinophil accumulation in pleural appeared significantly reduced in rats rendered diabetic 72 hr after alloxan [14]. In our study, we also found that eosinophil percentage in T2D was significantly lower than those with NGT or IGR. However, Kim et al found that with the accumulation of the total WBC count and differential WBC counts, the frequencies of diabetes, hypertension, obesity, dyslipidemia and metabolic syndrome increased in a Korea population [15]. Gulcan E et al evaluated the glucose tolerance status in patients with asthma bronchiale and suggested that disturbance of the glucose metabolism may occur in asthmatic patients and that the risk of diabetes mellitus may increase in these individuals [16]. Our results are not consistent with those of the two studies. The possibility may be the different study design and compositions of study subjects. Our study is based on general population in the community, whereas Kim enrolled participants who came for medical check-up and Gulcan E included asthmatic patients. The pre-existing factors involved in asthma pathogenesis may influence the association of glucose metabolism with eosinophil.

The mechanism of the relationship between eosinophil percentage and glucose metabolism has not been clarified. Some studies explained that decreased insulin level inhibited the recruitment of eosinophil [17]–[19]. However, we found that fasting serum insulin was significantly higher in T2D than those without T2D. There may be other mechanism for the decreased eosinophil in T2D, or the lower eosinophil is not a consequence of T2D but a risk factor for the incidence of T2D. Available biological data have strongly suggested that T2D is an inflammatory disease [1], [20]. Many of the immune cells including macrophage, neutrophil and eosinophil are involved directly or by producing inflammatory cytokines in pathology of chronic inflammation [21]. Eosinophil, one kind of immune cells, typically associates with allergy and parasitic infections, regulates the macrophage activation state in mammalian adipose tissue and may have an important role in metabolic homeostasis. In adipose tissue, eosinophil that migrates from the blood into adipose tissue, can produce IL-4 and IL-13, cytokines typically associated with the arm of the immune system that causes allergy but also protect against infection with parasites [8]. These two cytokines induce resident macrophages to become alternatively activated macrophages (AAMs) which improved control of glucose metabolism. Otherwise, downstream of IL-4 receptor α (IL-4Rα) is the nuclear hormone receptor peroxisome proliferator-activated receptor γ (PPARγ), and when activated, it inhibits the expression of genes that promote inflammation and protects against insulin resistance [22]. It is possible that the absence of eosinophil in adipose tissue, IL-4 and IL-13 concentrations are too low to counter the effects of infiltration of T cells that produce IL-6 and TNF-α [23].

However, the present study has several limitations. First, blood cortisol concentration was not tested in our study. Normal human eosinophil had glucocorticoid receptor which was capable of mediating biologic effects at physiologic hormonal concentrations [24]. In obesity, because of a down regulation of 11 β-HSD type 1 in the liver in parallel with an up regulation of the enzyme in adipose tissue [25], as well as because of increased plasma ACTH levels [26], cortisol production had a net increase. As a result, glucocorticoids may enhance eosinophil apoptosis through the intracellular glucocorticoid receptors [27]. Second, this was a cross-sectional study, and it could not exactly serve as a causal infer. Another limitation is the insulin resistance, which was evaluated by HOMA-IR index or serum insulin levels, instead of the hyperinsulinemic-euglycemic clamp. However, studies show that there is good correlation between estimates of insulin resistance derived from HOMA and from the euglycemic clamp [28].

In conclusion, we found that higher peripheral eosinophil percentage was independently associated with decreased risk of T2D and insulin resistance in middle aged and elderly Chinese. Insulin resistance played an important intermediate role in the association between eosinophil and impaired glucose metabolism.
